# Spontaneous regression of a growth hormone-secreting pituitary adenoma following thyroidectomy for toxic multinodular goiter with superior vena cava obstruction: Report of a rare case

**DOI:** 10.3892/mi.2025.286

**Published:** 2025-11-18

**Authors:** Bayar A. Qasim, Sardar H. Arif, Ashur Y. Izac, Halder J. Abozait, Rende S.A. Kochary

**Affiliations:** 1 Department of Medicine, College of Medicine, University of Duhok, Duhok 42001, Kurdistan Region, Iraq; 2 Department of Surgery, College of Medicine, University of Duhok, Duhok 42001, Kurdistan Region, Iraq

**Keywords:** acromegaly, multinodular goiter, superior vena cava obstruction, thyroidectomy, regression

## Abstract

Acromegaly is most commonly caused by a growth hormone (GH)-secreting pituitary adenoma; the spontaneous regression of such tumors is exceedingly rare. The present study reports the case of a 51-year-old male patient with long-standing acromegaly who developed a toxic multinodular goiter complicated by superior vena cava obstruction (SVCO). An initial evaluation revealed a pituitary macroadenoma measuring 19x17x16 mm, with elevated GH and insulin-like growth factor-1 (IGF-1) levels, consistent with acromegaly. The patient was treated with a somatostatin analog and metformin for glycemic control, followed by carbimazole in preparation for thyroidectomy. He subsequently underwent urgent total thyroidectomy for compressive symptoms due to SVCO. Post-thyroidectomy, he experienced marked clinical improvement, including the resolution of dyspnea, improved sleep quality, enhanced mood and partial regression of acromegaly symptoms. Biochemically, IGF-1 normalized within 6 months of total thyroidectomy, and an MRI demonstrated the progressive shrinkage of the pituitary adenoma, reducing to 8x6x5.5 mm at 1 year without neurosurgical intervention. Possible mechanisms for regression include perioperative pituitary apoplexy, spontaneous ischemia, or altered vascular dynamics following thyroidectomy. The present case report highlights the importance of reassessing pituitary adenomas following the treatment of coexisting endocrine or compressive disorders and suggests a potential role of vascular factors in pituitary tumor regression.

## Introduction

Acromegaly is a rare, yet serious disorder resulting from the chronic hypersecretion of growth hormone (GH) and insulin-like growth factor-1 (IGF-1), most commonly due to pituitary adenomas (1). Historically, patients with acromegaly were often viewed as attractions due to their unusual physical appearance (2). The disease develops insidiously, and clinical manifestations include the enlargement of the hands and feet, coarse facial features, fatigue, metabolic dysfunction and cardiovascular complications (3). Untreated acromegaly is associated with increased morbidity and mortality, primarily due to cardiometabolic and respiratory sequelae (4). The standard of care for GH-secreting adenomas is transsphenoidal resection, often followed by medical therapy, such as somatostatin analogs, dopamine agonists, or GH receptor antagonists, as well as radiotherapy when necessary (5).

Reports of the spontaneous regression of pituitary adenomas are exceedingly rare, particularly in hormone-secreting tumors. Regression, when it occurs, is typically attributed to pituitary apoplexy, ischemic infarction, or hemorrhage within the adenoma (6). Reports of GH-secreting adenomas undergoing spontaneous shrinkage are limited (7). Notably, thyroid disease, including multinodular goiter, is relatively common in acromegaly (78%) compared to other pituitary tumors (27%). However, the occurrence of toxic multinodular goiter with superior vena cava obstruction (SCVO) in such patients is unusual (8).

The present study describes the case of a middle-aged male patient with acromegaly who developed thyrotoxicosis due to a toxic multinodular goiter, complicated by SVCO. Following thyroidectomy, the patient experienced the regression of his pituitary adenoma and the normalization of his IGF-1 levels. To the best of our knowledge, such an association has not been previously reported in the literature.

## Case report

**Figure 1:**
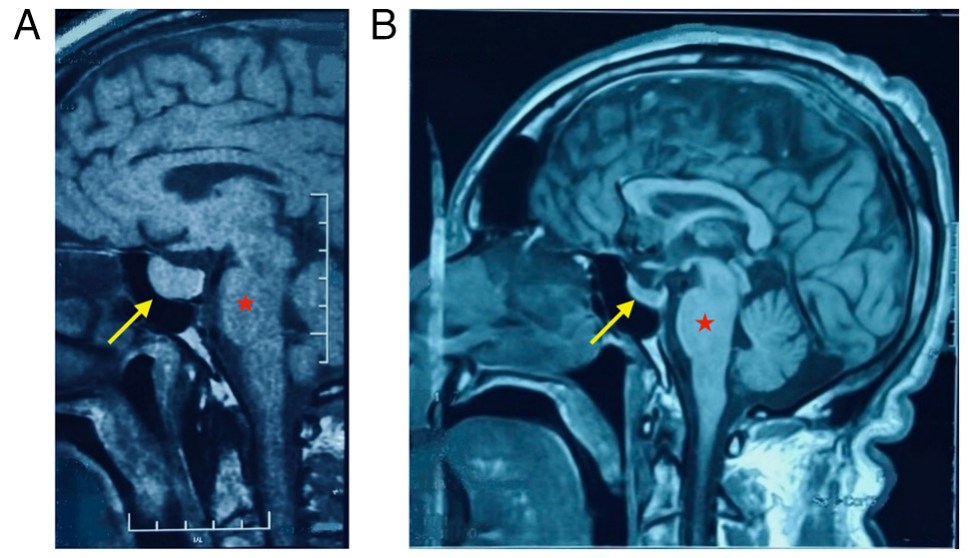
MRI of the pituitary gland in a 51-year-old male patient with acromegaly. (A) Prior to thyroidectomy, there was a pituitary macroadenoma (yellow arrow, 19x17x16 mm). (B) MRI illustrating the regression of the tumor to a microadenoma (yellow arrow, 8x6x5.5 mm) at 12 months following thyroidectomy. The red star indicates the pons.

The present study reports the case of a 51-year-old male patient with acromegaly who presented to the Azadi Teaching Hospital (affiliated with the College of Medicine at University of Duhok), Duhok, Iraq in November, 2023. He noted the progressive enlargement of his hands and feet, accompanied by changes in facial appearance, which began when he was 44 years of age. He required larger shoe sizes and clothing and his wedding band no longer fit. His family observed a gradual deepening of his voice. The patient experienced easy fatigability; however, he had no headaches, diaphoresis or fever, and did not experience any weight gain. He was yet to meet a specialist at this time. Subsequently, at the age of 46 years, he developed a worsening shortness of breath, poor sleep quality and frequent nocturnal dyspnea relieved by sitting upright. He also reported excessive daytime somnolence and irritability, with no cough, chest pain or palpitations. He was first evaluated for acromegaly at the age of 48 years through laboratory and imaging analyses. The GH suppression test revealed high GH levels (62.5 ng/ml) that could not be suppressed, with levels remaining unaltered at 1 and 2 h following a 75 g oral glucose tolerance test (in a normal response, GH is suppressed to <1 ng/ml). His IGF-1 level was 467 ng/ml [2.33-fold above the upper limit of the normal range; reference range (RR), 84-200 ng/ml)] and his HbA1c level was 6.4% (RR, <5.7%). A pituitary MRI revealed a pituitary macroadenoma measuring 19x17x16 mm on the right side of anterior pituitary gland (Fig. 1A). Treatment was initiated with a somatostatin analog (Sandostatin LAR at 30 mg every 4 weeks) and metformin (750 mg once daily) for uncontrolled blood glucose levels.

**Figure 2:**
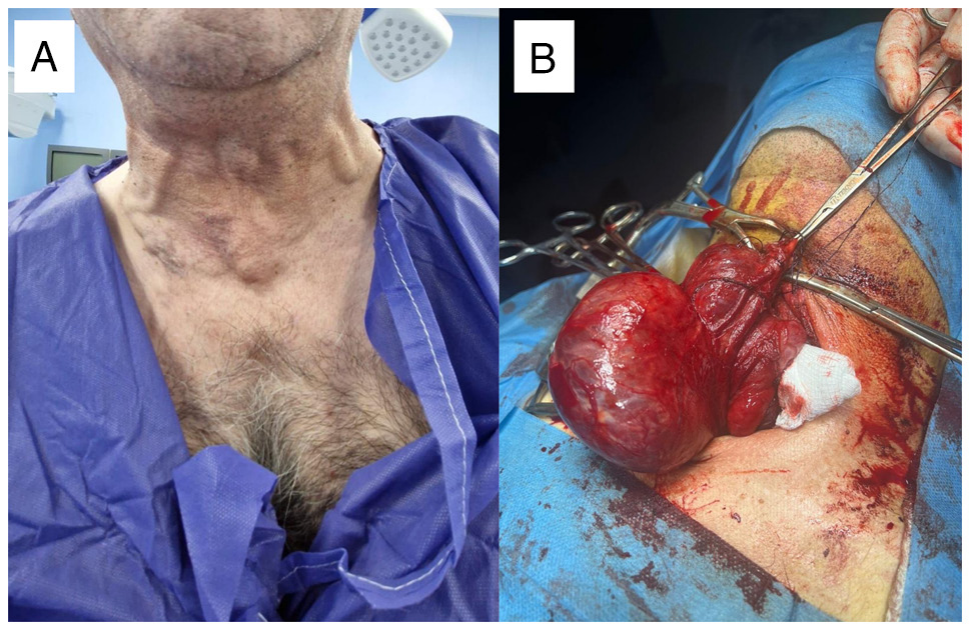
(A) Distended neck veins in a 51-year-old male patient with retrosternal goiter and superior vena cava obstruction. (B) Intraoperative image of a 260-g thyroid gland; the right lobe measured 110x70x50 mm with a 65-mm spherical nodule while left lobe measured 60x40 mm.

**Figure 3:**
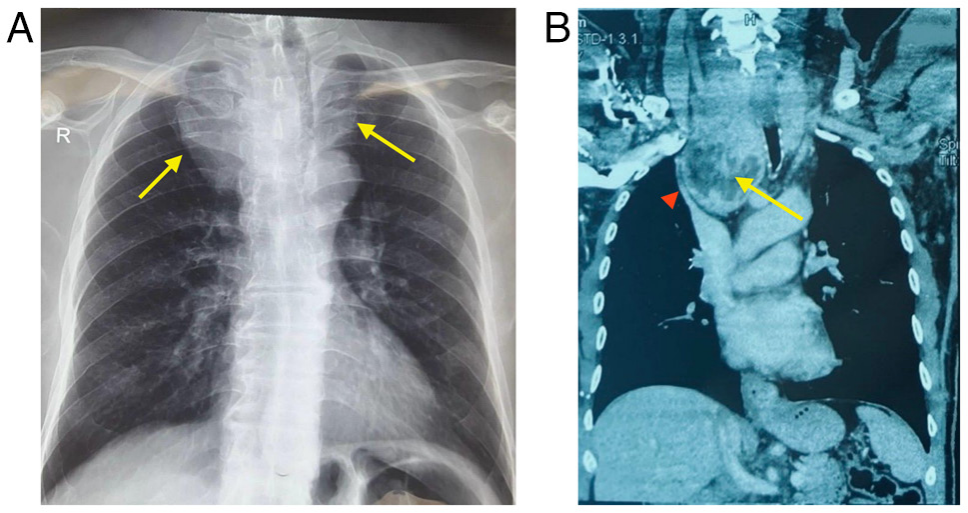
(A) Chest radiograph illustrating a retrosternal mass (yellow arrows). (B) Computed tomography scan of the chest illustrating a heterogenous thyroid mass the is extending retrosternally (yellow arrow), compressing superior vena cava (red arrowhead) and pushing trachea to the left side.

**Figure 4:**
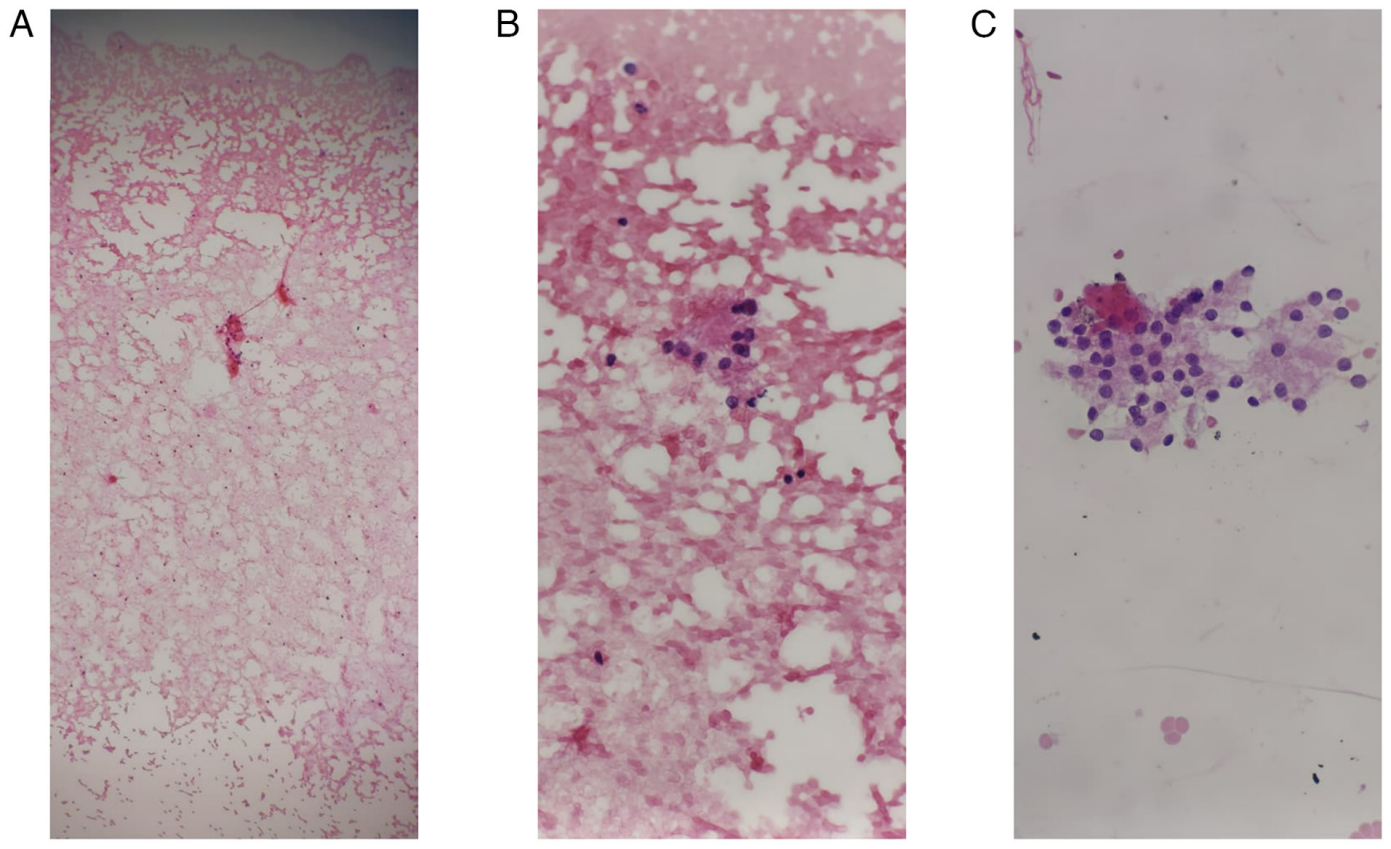
Fine-needle aspiration cytology smears from a 51-year-old male with retrosternal goiter illustrating (A and B) groups of follicular epithelial cell in a colloid-rich background [H&E staining; magnification: (A) x5, (B) x20] with (C) scattered hemosiderin-laden macrophages (H&E staining; magnification x20). Cytomorphological features are consistent with a benign thyroid lesion (Thy 2).

At the age of 49 years, a physical examination revealed coarse facial features, a prominent nose, interdental spacing and poor dentition. His thyroid gland was asymmetrically enlarged, and distended neck veins were noted (Fig. 2A). His extremities revealed large hands and feet. A laboratory investigation revealed primary hyperthyroidism with a normal short-Synacthen test result (Table I). However, the short-Synacthen test may yield false-negative results in the early central adrenal insufficiency. A thyroid ultrasonography revealed an enlarged thyroid gland with a large nodule in the left lobe (TIRADS 3). Neck and chest imaging revealed a large thyroid mass extending retrosternally and compressing the superior vena cava (Fig. 3). An ultrasound-guided fine-needle aspiration cytology (FNAC) of the thyroid gland confirmed multinodular goiter (Fig. 4). The hematoxylin and eosin staining illustrated in Fig. 4 was performed by the Pathology Laboratory at VIN Specialized Medical Laboratories in Duhok, Iraq. The patient was commenced on carbimazole therapy at 40 mg daily, along with propranolol 40 mg daily, to prepare for total thyroidectomy. Following 4 months of carbimazole dose-adjustments, his thyroid function test results were normalized [thyroid-stimulating hormone (TSH), 1.2 µIU/ml; free T4, 12.8 pmol/l)] and he was scheduled for surgery.

The patient underwent an urgent total thyroidectomy at the age of 50 years to relieve SVCO. The removed thyroid weighed 260 g, with the right lobe measuring 110x70x50 mm with a 65-mm spherical nodule, while the left lobe was 60x40 mm (Fig. 2B). The post-thyroidectomy course was uneventful. Immediately following surgery, the patient reported a marked improvement in dyspnea, the resolution of orthopnea and an improved sleep quality, with no acute complications or visual disturbances following surgery. Over the following months, he noted an improvement in his mood, energy levels, and a partial reduction in the size of his hands, feet and facial features. Follow-up laboratory tests revealed the normalization of thyroid function (TSH, 3.02 µIU/ml; free T4, 20.7 pmol/l) with appropriate levothyroxine replacement (75 mcg/day) that required dose adjustment over the course of 5 months until a stable dose of 125 mcg/day. Notably, his IGF-1 levels gradually declined and normalized (84 ng/ml) within 6 months post-thyroidectomy. An MRI of the pituitary revealed a marked shrinkage in the size of the pituitary adenoma, which decreased from 19x17x16 mm prior to the thyroidectomy (Fig. 1A) to 14x11x9 mm at 5 months post-thyroidectomy, and further to 8x6x5.5 mm at 12 months post-thyroidectomy (Fig. 1B). At the 18-month follow-up, the patient remained asymptomatic, with well-controlled diabetes (HbA1c 4.7%) on metformin (750 mg once daily), and stable thyroid and pituitary function on levothyroxine and somatostatin analog therapy (Table I). He continues follow-up with an endocrinologist; no neurosurgical intervention has been required and the planned transsphenoidal resection of the pituitary adenoma was cancelled.

## Discussion

The present case report illustrates the rare coexistence of acromegaly due to a GH-secreting pituitary adenoma with toxic multinodular goiter causing SVCO. Although thyroid nodular disease is common among patients with acromegaly, the development of thyrotoxicosis complicated by vascular compression is unusual (8). The most notable feature of the case presented herein was the unexpected regression of the pituitary adenoma following thyroidectomy.

The regression of pituitary adenomas, particularly GH-secreting tumors, is rare and typically attributed to ischemic infarction or pituitary apoplexy (7). In the patient in the present study, a single or a combination of mechanisms may explain the observed regression. The first possibility is the spontaneous, natural shrinkage of a macroadenoma, which has been described in large tumors with limited vascular supply (9). The second mechanism is pituitary apoplexy induced by sudden perioperative hemodynamic fluctuations, as the vasculature of pituitary adenomas is fragile and susceptible to ischemic or hemorrhagic events (9). A third contributing factor may be elevated levels of thyroid hormones, which enhance GH/IGF-1 secretion and metabolic activity, potentially exacerbating acromegaly severity, with resolution following thyroidectomy supporting this hypothesis (10). The last and most intriguing hypothesis is that the massive multinodular goiter may have compromised carotid circulation and hypophyseal arteries supplying the pituitary gland, with surgical removal altering vascular dynamics and precipitating ischemic changes within the adenoma. While this mechanism has not specifically been discussed in the literature, large goiters with carotid compression are known to cause ischemic stroke in rare circumstances (11).

The regression of symptoms of acromegaly and the normalization of IGF-1 levels following thyroidectomy support the hypothesis of treatment-tumor infarction rather than spontaneous shrinkage. The biochemical normalization may also be attributed to somatostatin analogue. If the patient had been subjected directly to transsphenoidal surgery, the patient may have undergone an unnecessary invasive procedure. Instead, the improvement following thyroidectomy highlights how endocrine and vascular interactions can influence tumor biology. An Italian multicenter study revealed that in patients with acromegaly, non-toxic nodular goiter was found in 40% of cases, while toxic nodular goiter was found in 13.4% of cases (8). A previous case report described the anesthetic challenges encountered in patients with acromegaly when thyroid disease compromises the airway (12). Another case report highlighted the occurrence of acromegaly with multinodular goiter in a patient with McCune-Albright syndrome, where there was spontaneous normalization of thyroid function and reduction of pituitary tumor size with somatostatin analog therapy (13). A different case report documented the spontaneous shrinkage of a pituitary adenoma in a patient with hypothyroidism, with the authors of that study proposing the cause to be autoimmune lymphocytic hypophysitis (14). No prior studies have documented the shrinkage of GH-secreting tumors following thyroidectomy.

In conclusion, the present case report describes a rare instance of acromegaly due to a GH-secreting pituitary adenoma coexisting with toxic multinodular goiter complicated by SVCO. Following urgent thyroidectomy, the patient experienced clinical and biochemical improvement with the regression of symptoms of acromegaly, the normalization of IGF-1 levels and the radiological shrinkage of the adenoma, without the need for neurosurgical intervention. The present case report highlights the potential role of vascular mechanisms in pituitary tumor biology and emphasizes the importance of carefully reassessing of pituitary lesions before proceeding with definitive surgical management.

## Figures and Tables

**Table I:** Hormonal profile of the patient prior to and following thyroidectomy.

Hormone	Prior to surgery	Following surgery	Reference range	Interpretation
GH (ng/ml)	62.5	8.42	0.4-10	Normalized
IGF-1 (ng/ml)	467 (2.33x ULN)	108	84-200	Normalized
Prolactin (ng/ml)	13.44	12.38	4.04-15.2	Normal
TSH (µIU/ml)	<0.005	3.02	0.27-4.2	Restored
Free T4 (pmol/l)	54.15	20.7	12-22	Normalized
LH (mIU/ml)	3.56	3.4	1.7-8.6	Normal
FSH (mIU/ml)	2.4	2.9	1.5-12.4	Normal
Testosterone (nmol/l)	11.9	14.6	8.64-29	Normal
ACTH (pg/ml)	42	20	7.2-63.3	Normal
Cortisol^a^ (nmol/l)	393 → 667 → 675	262 → 682 → 679	Baseline, >180; 30 min and 60 min >500-550	Normal adrenal response

^a^Cortisol levels shown at baseline, and at 30- and 60-min post-Synacthen test values. GH, growth hormone; ULN, upper limit of normal range; IGF-1, insulin-like growth factor 1; TSH, thyroid-stimulating hormone; LH, luteinizing hormone; FSH, follicle-stimulating hormone; ACTH, adrenocorticotropic hormone.

## Data Availability

The data generated in the present study may be requested from the corresponding author.
